# Human rights principles in developing and updating policies and laws on mental health

**DOI:** 10.1017/gmh.2016.5

**Published:** 2016-04-12

**Authors:** M. Schulze

**Affiliations:** Consultant, Human Rights Consultant, Vienna, Austria

**Keywords:** CRPD, human rights, mental health, policy and systems

## Abstract

The World Health Organization's Mental Health Action Plan 2013–2020 stipulates human rights as a cross-cutting principle (WHO, 2013) and foresees global targets to update policies as well as mental health laws in line with international and regional human rights instruments. The international human rights agreements repeatedly refer to health, including mental health. The most pertinent provisions related to mental health are enshrined in the 2006 Convention on the Rights of Persons with Disabilities (CRPD), which sets out human rights in an accessible and inclusive fashion to ensure the equal participation of persons with disabilities. The inconclusive description of disability in the treaty overtly refers to ‘mental impairment’ as part of an explicitly evolving understanding of disability. This text sketches some of the underlying concepts as they apply to the realm of mental health: non-discrimination of persons with disabilities and measures that should be taken to ensure accessibility in a holistic understanding; removal of social and attitudinal barriers as much as communication and intellectual barriers but also institutional hurdles. The CRPD's paradigm shift away from framing disability mainly through deficits towards a social understanding of disability as the result of interaction and focusing on capacity is the core on which the provision of mental health services at community level to enable participation in society shall be ensured. Questions of capacity, also to make decisions and the possible need for support in so doing, are sketched out.

## Introduction

Mental health has seen increased attention at various policy levels over the past decade(s). At the global level, the WHO Action Plan is the latest milestone aimed at strengthening mental health for all. The coming into force of the Convention on the Rights of Persons with Disabilities (CRPD) in 2008 has added to the momentum of human rights aspects in this field, providing an opportunity to reinforce pertinent efforts and revisit some issues, particularly from a multi-disciplinary point of view. This has been underscored by the WHO's World Report on Disability (WHO/World Bank, 2011) – the first of its kind – as well as the subsequent Action Plan on Disability (WHO, 2014). The aim of this piece is to sketch the CRPD's core concepts and principles in relation to mental health and human rights based approach thereto.

## Principles and rights

### Non-discrimination

#### The obligation of non-discrimination

‘Both de jure and de facto discrimination against persons with disabilities have a long history and take various forms. They range from invidious discrimination, such as the denial of educational opportunities, to more ‘subtle’ forms of discrimination such as segregation and isolation achieved through the imposition of physical and social barriers,’ observed the Committee on Economic, Social and Cultural Rights (CESCR, 1994). The observation certainly still holds true for most persons with disabilities in most countries. This opinion of the Committee formed a corner stone of the negotiations of the CRPD, which states clearly that discrimination on the basis of disability ‘means any distinction, exclusion or restriction on the basis of disability which has the purpose or effect of impairing or nullifying the recognition, enjoyment or exercise, on an equal basis with others, of all human rights and fundamental freedoms in the political, economic, social, cultural, civil or any other field. It includes all forms of discrimination, including denial of reasonable accommodation,’ (Article 2 CRPD).

Note that this – legal – definition includes discrimination at the above-mentioned more subtle levels, such as ‘indirect’ discrimination as well as reasons that add to the severity of experiencing exclusion: multiple and aggravated forms of discrimination (PP (p) CRPD). The notion of ‘reasonable accommodation’ is defined as ‘necessary and appropriate modification and adjustments not imposing a disproportionate or undue burden, where needed in a particular case, to ensure to persons with disabilities the enjoyment or exercise on an equal basis with others of all human rights and fundamental freedoms, (Article 2 CRPD).’

#### The ‘non-definition’ of disability: fix society, not people!

The CRPD negotiators perused more than 50 definitions of impairment and disability, respectively (Schulze, 2010). The conclusion was that the ‘evolving’ nature, i.e. concept, of disability (PP (e) CRPD) makes a definite delineation impossible, hence an open description that may be labeled a ‘non-definition’ was agreed: ‘Persons with disabilities include those who have long-term physical, mental, intellectual or sensory impairments, which in interaction with various barriers may hinder their full and effective participation in society on an equal basis with others, (Article 1 CRPD).’ In addition to the explicit mention of ‘mental impairment,’ note the emphasis placed on other barriers, particularly those of an ‘attitudinal’ nature, which ‘in interaction with environmental barriers’ negate the inclusion of and accessibility for persons with disabilities (PP (e) CRPD). The importance of such external factors is underscored by data showing the likelihood of persons with mental impairment being subject to violence and abuse as well as the frequency with which they are deprived of their human rights, particularly their civil and political rights (WHO, 2010*b*).

The shift of focus to the ‘others’ – also as the cause of a disabling environment – necessitates a clear move away from a purely medical description, a deficit-only description of impairment and persons with disabilities, respectively. The bio-psycho-social model enshrined in the International Classification on Functioning, Disability and Health (ICF) receives new impetus in the CRPD's aim to fix attitudes rather than perceived impairments and ‘deficits,’ respectively. The ICF ‘views disability as a combination of various individual, institutional and societal factors that define the environment within which a person with impairment exists,’ (Trani, 2011). It makes multi-disciplinary assessment of persons with disabilities, including those with mental impairments, a necessity in order to adequately capture the environmentally and particularly socially related barriers. The paradigm shift away from purely medical parameters to including social aspects (Shakespeare, 2006; Trani, 2011) does not only call for training of medical staff (Article 25 (d) CRPD) but also comprehensive awareness raising to, inter alia, ‘combat stereotypes, prejudices and harmful practices relating to persons with disabilities, including those based on sex and age, in all areas of life (Article 8 CRPD).’

#### Not merely planning ramps: accessibility

Overcoming the more ‘subtle’ forms of discrimination and, importantly, the manifold ways in which the agency of persons with disabilities is structurally impaired (Farmer, 2005), requires a focus on the social as well as attitudinal barriers that the mainstream plans, builds and sustains by way of stereotypes, prejudices and outright hate crime (OSCE, 2012).

Another accessibility angle that the CRPD emphasizes: communication (Article 2, CRPD). Ensuring access to information and communication for hearing and visually impaired persons. Importantly, the CRPD emphasizes the means and modes of communication other than verbal, highlighting augmentative and other devices. Note that orientation in buildings, including hospitals, also falls within this category. The paths to communication, also for non- and semi-verbal persons are manifold, as the CRPD clearly states. Supported communication embodies a further, important, dimension: intellectual accessibility. While primarily tailored for persons with learning difficulties or intellectual impairment, respectively, Easy-to-Understand formats have potential for many, including those in distress. Also, supports such as personal assistants or refuge spaces can be grounded in the Convention's broad concept of ‘accessibility.’

The most established dimension of accessibility is the physical or architectural one: ensuring that mobility impaired persons – as well as elderly people and those using prams, rollers and other mobility devices – have access to all premises. A human rights based approach highlights that persons with disabilities have largely been deprived of adequate opportunities to achieve good quality education and possibilities to earn sufficient income: economic accessibility or affordability, respectively, is therefore of great importance. Finally, institutional accessibility shall ensure that structural barriers, which limit the opportunities for persons with disabilities to participate on an equal basis with others, be removed: in planning, programing, etc.

Access to insurance is frequently denied on discriminatory grounds related to disability and impairment, respectively, including mental impairment diagnosis. The CRPD requires States to ensure provisions in a fair and reasonable manner where national laws foresee health and life insurance – Article 25 (e) CRPD. Finally, equality – and therewith non-discrimination – shall be guaranteed by way of reasonable accommodation (Article 5 (3) CRPD) as defined in Article 2 (see above).

### Right to health

Clearly, all human rights obligations have relevance for the realm of mental health, an obvious starting point is the right to health itself and those provisions that refer to closely related fields such as rehabilitation (CESCR, 2004). The ‘right to health embraces a wide range of socio-economic factors that promote conditions in which people can lead a healthy life, and extends to the underlying determinants of health, such as food and nutrition, housing, access to safe and potable water and adequate sanitation, safe and healthy working conditions, and a healthy environment (CESCR, 2004).’

The CRPD enshrines the right to health as follows (note that the notion of ‘physical and mental health of the Covenant on Economic, Social and Cultural Rights (CESCR), Article 12 is reframed in the context of the CRPD's Article 25 to right to health).’ See further: Schulze (2010), Understanding the Convention on the Rights of Persons with Disabilities. See Article 12 Convention on the Elimination of All Forms of Discrimination Against Women (CEDAW) and Article 24 Convention on the Rights of the Child (CRC) for further provisions on the right to health. Note the application for stateless persons and asylum seekers: OHCHR/WHO Fact Sheet 31, Page 20.): Box 1.

Box 1. Article 25 Convention on the Rights of Persons with Disabilities

States Parties recognize that persons with disabilities have the right to the enjoyment of the highest attainable standard of health without discrimination on the basis of disability. States Parties shall take all appropriate measures to ensure access for persons with disabilities to health services that are gender-sensitive, including health-related rehabilitation. In particular, States Parties shall:

(a) Provide persons with disabilities with the same range, quality and standard of free or affordable health care and programmes as provided to other persons, including in the area of sexual and reproductive health and population-based public health programmes;
(b) Provide those health services needed by persons with disabilities specifically because of their disabilities, including early identification and intervention as appropriate, and services designed to minimize and prevent further disabilities, including among children and older persons;
(c) Provide these health services as close as possible to people's own communities, including in rural areas;
(d) Require health professionals to provide care of the same quality to persons with disabilities as to others, including on the basis of free and informed consent by, inter alia, raising awareness of the human rights, dignity, autonomy and needs of persons with disabilities through training and the promulgation of ethical standards for public and private health care;
(e) Prohibit discrimination against persons with disabilities in the provision of health insurance, and life insurance where such insurance is permitted by national law, which shall be provided in a fair and reasonable manner;
(f) Prevent discriminatory denial of health care or health services or food and fluids on the basis of disability.

As the Special Rapporteur on the Right to Health observed in a pertinent report on mental health and disability: ‘(The right to health) is a right to facilities, goods, services and conditions that are conducive to the realization of the highest attainable standard of physical and mental health. States should ensure facilities, goods, services and conditions for persons with mental disabilities so they may enjoy the highest attainable standard of health (Special Rapporteur, 2005).’ Specifically, the right to health entails freedoms such as non-discrimination (OHCHR/WHO, 2008; CESCR, 2004) and the right of control over one's health, body and physical as well as mental integrity (see also, below section Participation and peer support), among others.

Importantly, the provision of health services, particularly for mental health, shall be community based (Articles 19, 25 (c) & 26 (1)(b) CRPD; WHO, 2010*a*). As the Special Rapporteur states community based services are ‘conducive to health, dignity and inclusion,’ and – ultimately – ‘unnecessary institutionalization can be avoided (Special Rapporteur, 2005).’ Services should include medication, physiotherapy, ambulatory services, residential facilities, programs to maximize the independence and skills of persons with disabilities (Articles 9 (2 (e)) & 24 (3) CRPD). The WHO Quality Rights Toolkit emphasizes the importance of closing down large-scale facilities and improving health service delivery based on the CRPD (WHO, 2012).

### Autonomy and the freedom to make one's own choices

The CRPD's first general principle states the ‘respect for inherent dignity, individual autonomy including the freedom to make one's own choices, and independence of persons,’ (Article 3 (a) CRPD) The will and preferences of persons with disabilities generally and specifically those with mental impairments have not been at the forefront of health care provision in most countries (Special Rapporteur, 2005). Institutional rigidity, liability considerations and a lack of knowledge are among many factors that have contributed to and resulted in a perception of persons with mental and similar impairments as largely ‘incapable’ of making decisions related to most aspects of their health – and sometimes their life. Laws and directives as well as routine and practice sustain the underlying assumptions. However, ‘people once thought incapable of making decisions for themselves have shattered stereotypes by showing that they are capable of living independently if provided with appropriate legal protections and supportive services. Moreover, many people once thought permanently or inherently limited by a diagnosis of major mental illness have demonstrated that full recovery is possible (Special Rapporteur, 2005).’

In addition to informed consent (Article 25 (d) CRPD), the treaty foresees consent in order to ensure the enjoyment of the freedom from torture, cruel, inhumane and degrading treatment and punishment (Article 15 (1) CRPD). Furthermore, Article 12 enshrines ‘Equal recognition before the law,’ which entails both the right to legal capacity as well as the right to act that legal capacity. Contrary to what some debates around legal capacity imply, this provision is not unique to persons with disabilities, it has an important predecessor in the Convention on the Elimination of All Forms of Discrimination Against Women, which equally enshrines legal capacity and – importantly – the right to act such capacity (Article 15 CEDAW). The advances made as well as the progress still awaiting law and practice in most countries in terms of equality for women may serve as an indicator for both the potential and the paradigm-shift in ensuring legal capacity for all persons with disabilities.

The CRPD requires a move away from substituted decision-making to models of supported decision-making (Article 12 CRPD; CRPD Committee, 2014*a*). The holistic understanding of assistance and support, which underlies the CRPD pulls into question a lot of the assumptions, customs and practices vis-à-vis persons with mental and similar impairments. The CRPD questions the legitimacy of institutions such as guardianship, which significantly limit the legal capacity and the practical aspect of acting on that capacity. The concept of supported decision-making has its challenges; the vibrant discussion it has caused points to a variety of factors, not least the importance of that debate (CRPD, 2014*a*).

The broad understanding of support and assistance, the importance of persons with disabilities providing instructions and having a right to see their will and preferences applied is particularly well enunciated elsewhere in the treaty: see the provision on the right to vote: ‘Guaranteeing the free expression of the will of persons with disabilities as electors and to this end, where necessary, at their request, allowing assistance in voting by a person of their own choice (Article 29 (a) (iii) CRPD).’

‘The loss of control over one's life that follows from the deprivation of legal capacity has negative effects on the person's sense of the self (Dhanda, 2007).’ What is more: ‘a label of incompetence can easily become a self-fulfilling prophecy. If not given any opportunities to make decisions, how can we learn to do so and take responsibility for our choices? (Human Rights Commissioner, 2012).’ Learning to make decisions, to receive support on the path to decision-making is part of ‘unlearning’ how the legal capacity of persons with disabilities has been substituted by others who mean(t) well. Part and parcel of that process as well as state-of-the-art practice has to be the right to make a mistake and a culture that enables a certain level of risk; at the very least there has to be a profound debate over how risk is assessed. Clearly, a culture where ‘no mistake’ is foreseen is incompatible with the CRPD. Ultimately, it is part of everyone's dignity to take risks and learn from mistakes: the ‘dignity of risk’ (Deegan, 1996).

Interestingly, the projection of incapability fails to acknowledge the range of support, which ‘chronically normal persons’ (Deegan, 1996) practice on a daily basis. Requests for information, seeking counsel both informal and through consultants, management and policy decisions based on a plethora of sources, decisions by chief executives and government leadership informed by entire armies of advisers are standard. While inherently different in setting and choice of support, the underlying method is no different to the thrust of supported decision-making. Ultimately, the idea of inclusion is not just that we enable persons with disabilities to be equal, to enjoy the same rights and freedoms – as they should have already for a long time. The challenge lies in asking ourselves what the mainstream does routinely and how persons with disabilities, because they are labeled ‘different’ and ‘special’ and ‘needy’ are missing out. Clearly, impairment, including mental health, is not to be viewed as a static but a fluid concept, not least when it comes to the discussion over legal capacity and risk taking, respectively.

A major challenge on the path to ensuring autonomy and free choices is a culture of liability that permeates institutions and their processes as well as procedures. Leverage for a culture of risk will have to be expanded to enable decision-making processes for persons with disabilities on an equal basis with others.

### Right to privacy

‘States Parties shall protect the privacy of personal, health and rehabilitation information of persons with disabilities on an equal basis with others,’ Article 22 (2) CRPD responds to the various paternalistic practices that tend to share the confidential information of persons with disabilities more widely than other patients’. This includes the tendency to talk to relatives and assistance very freely, frequently circumventing the relaying of information to the actual patient. An important dimension is the inaccessibility of forms and procedures, which imply a necessity to lower standards of confidentiality rather than revise them in light of accessibility standards. Note that the CRPD explicitly calls for the abolishing and amendment of customs and practices that discriminate on the basis of disability and impairment, respectively (Article 4 (1)(b) CRPD).

### Freedom from inhuman treatment and protection against violence

The adoption of the CRPD has brought highly overdue focus to severe human rights violations directed at persons with disabilities. Importantly, violence and other human rights violations against the physical and mental integrity of persons with disabilities occur not only in institutions but also in private settings; violations are considered to be happening widely and due to the taboo nature of violence more generally exacerbated by the stigma associated with disability (WHO, 2010*b*).

The obligations in the CRPD are closely connected to the freedom from inhuman treatment as enshrined in the Convention Against Torture (CAT) and the Covenant on Civil and Political Rights (CCPR). Importantly, the provisions on freedom from violence and inhuman treatment in the CRPD are placed between legal capacity (equal recognition before the law, Article 12), Access to Justice (Article 13) and the protection of integrity (Article 17). Liberty and security of persons with disabilities includes the right not to be deprived of one's liberty unlawfully or arbitrarily, and that ‘a disability shall in no case justify a deprivation of liberty,’ (Article 14 (1)(b) CRPD; CRPD Committee, 2014*b*).

Note that in addition to substantive protections, this set of provisions also foresees the obligation to establish a monitoring entity: ‘In order to prevent the occurrence of all forms of exploitation, violence and abuse, States Parties shall ensure that all facilities and programmes designed to serve persons with disabilities are effectively monitored by independent authorities,’ Article 16 (3) CRPD. This requirement should not be mistaken with the extensive national monitoring provisions in Article 33 CRPD as well as international requirements set out in Article 34 ff CRPD.

A provision with high relevance to mental health, particularly where abuse, violence and other human rights violations have occurred is Article 16 (4) CRPD: ‘States Parties shall take all appropriate measures to promote the physical, cognitive and psychological recovery, rehabilitation and social reintegration of persons with disabilities who become victims of any form of exploitation, violence or abuse, including through the provision of protection services.’ The recovery process is to be enabled at community level: ‘Such recovery and reintegration shall take place in an environment that fosters the health, welfare, self-respect, dignity and autonomy of the person and takes into account gender- and age-specific needs.’

### Participation and peer support

The negotiation process of the CRPD was defined by the high-level participation of persons with disabilities, both as members of delegations as well as civil society representatives (Sabatello, 2013). As a result, the CRPD spells out an obligation to involve persons with disabilities and their representative organization, in all policy-making processes – Article 4 (3) CRPD. While primarily directly at the State – as the responsible entity for promoting, protecting and ensuring human rights obligations – there are various provisions, which highlight corresponding obligations to ensure participation by third parties, including, e.g. hospitals, etc. Participation is a general principle of the treaty (Article 3) and resonates throughout the Convention as much as the principle of inclusion, which also necessitates participation.

Peer support is another noteworthy approach the CRPD explicitly foresees, particularly in the realm of rehabilitation (Article 26): ‘to attain and maintain a maximum independence, full physical, mental, social and vocational ability, and full inclusion and participation in all aspects of life,’ effective measures, including peer support shall be taken. Rehabilitative measures shall be provided in the community (WHO, 2010*a*), they shall, among others, ensure access to education, which also foresees the teaching of life and social development skills to enable full participation in the community (Article 24).

## The process of aligning mental health policies and law with international standards

As the World Health Organizations’ Resource Book on Mental Health, Human Rights and Legislation suggests that the review of international human rights obligations forms part of the review process of mental health laws as stipulated by the WHO Mental Health Action Plan. Given the rate of ratifications of the CRPD the vast majority of countries are now under an obligation to base their mental health laws on earlier human rights treaties as well as the CRPD. Familiarizing the various stake-holders (WHO, 2005, 2012) of the amendment process with the CRPD's key concepts and principles is paramount to ensure that its standards are reflected. The CRPD itself foresees training as a general obligation – Article 4 (1)(i) CRPD – as well as specifically for medical staff: Article 25 (d) CRPD.

A frequent lacuna of legislation in the realm of disability, also mental health, is data: in response, the CRPD explicitly foresees the collection of data and statistics: Article 31. It may be necessary to significantly revamp the collection of data, which when existent often relies on the outdated medical model of disability rather than the bio-psycho-social model as outlined, among others, in the ICF.

The process of drafting or redrafting a mental health policy and legislation has to involve persons with disabilities with experience in that field. Unusual for some it proves time and again that after a period of initial adjustment, the quality of the discussion benefits greatly from that involvement. Not surprisingly, potential hurt between professionals and self-advocates may need to be addressed. Those institutions familiar with recovery, trialogue and similar approaches to mental health can be of great assistance in those initial phases.

The potential of such processes also as opportunities to raise awareness about the importance of mental health as well as reducing stigma, should not be underestimated. Enabling persons with disabilities to speak up for themselves and be taken seriously as participants in such processes is immeasurable. Note that the CRPD has a detailed provision on awareness-raising enshrined in Article 8.

## Conclusion

The CRPD enshrines principles that many have worked with long before it was negotiated and it formulates rights on which many have based their work for a long time. Notwithstanding, many persons with disabilities, including the majority of persons with mental impairments, have been deprived of those principles and rights. The WHO Mental Health Action Plan's goal to bring legislation and practice in line with the CRPD accordingly has to be more than aspiration. Implementation of this human rights treaty requires both the amendment and expansion of legislation but importantly also of practice, particularly such practice that discriminates and that neglects (Article 4 Para 1 CRPD).

The treaty's aspirations were largely framed by self-advocates, including persons with mental impairments. It is the solidarity among and between persons with different impairments that brought the negotiations over the finishing line. In this vein, a quote from the late Lex Grandia, inaugural President of the World Federation of the Deaf-Blind:

“WOULD YOU WANT THIS TO HAPPEN TO YOU?

• Wouldn't you have the feeling that you have lost your dignity and want it back?”
• Wouldn't you feel your integrity has been violated?
• Wouldn't you want to have support in making decisions without being taken over and to ask for help without being seen any the less for it?
• Wouldn't you want to maintain your inherent dignity and be supported to make your own decisions?” (Grandia, 2013).
